# Proanthocyanidins Prevent High Glucose-Induced Eye Malformation by Restoring *Pax6* Expression in Chick Embryo

**DOI:** 10.3390/nu7085299

**Published:** 2015-08-07

**Authors:** Rui-Rong Tan, Shi-Jie Zhang, Yi-Fang Li, Bun Tsoi, Wen-Shan Huang, Nan Yao, Mo Hong, Yu-Jia Zhai, Zhong-Fu Mao, Lu-Ping Tang, Hiroshi Kurihara, Qi Wang, Rong-Rong He

**Affiliations:** 1 Anti-stress and Health Research Center, Pharmacy College, Jinan University, Guangzhou 510632, China; E-Mails: tanruirong@foxmail.com (R.-R.T.); zsj19891122@gmail.com (S.-J.Z.); liyifangkele@sina.com (Y.-F.L.); amytsoi@163.com (B.T.); 15692001436@163.com (W.-S.H.); hongmo12345@163.com (M.H.); kapono@126.com (Y.-J.Z.); maozhongfu2006@126.com (Z.-F.M.); tangluping@sina.cn (L.-P.T.); hiroshi_kurihara@163.com (H.K.); 2 Institute of Clinical Pharmacology, Guangzhou University of Chinese Medicine, Guangzhou 510006, China; 3 Guangdong Research Institute of Traditional Chinese Medicine Manufacturing Technology, Guangzhou 510095, China; E-Mail: nanyao2000@126.com

**Keywords:** proanthocyanidins, hyperglycemia, eye malformation, oxidative stress, *Pax6*, chick embryo

## Abstract

Gestational diabetes mellitus (GDM) is one of the leading causes of offspring malformations, in which eye malformation is an important disease. It has raised demand for therapy to improve fetal outcomes. In this study, we used chick embryo to establish a GDM model to study the protective effects of proanthocyanidins on eye development. Chick embryos were exposed to high glucose (0.2 mmol/egg) on embryo development day (EDD) 1. Proanthocyanidins (1 and 10 nmol/egg) were injected into the air sac on EDD 0. Results showed that both dosages of proanthocyanidins could prevent the eye malformation and rescue the high glucose-induced oxidative stress significantly, which the similar effects were showed in edaravone. However, proanthocyanidins could not decrease the glucose concentration of embryo eye. Moreover, the key genes regulating eye development, *Pax6*, was down-regulated by high glucose. Proanthocyanidins could restore the suppressed expression of *Pax6*. These results indicated proanthocyanidins might be a promising natural agent to prevent high glucose-induced eye malformation by restoring *Pax6* expression.

## 1. Introduction

Gestational diabetes mellitus (GDM) is a maternal state with a proven higher fetal malformation rate [1]. Infants born to women with GDM are at increased risk of adverse perinatal outcomes, such as congenital anomalies, macrosomia leading to birth trauma [2], hypoglycaemia [3], respiratory distress [4], polycythaemia jaundice [5], *etc*. Among these abnormalities, eye malformation has drawn great attention. Previous studies showed that maternal type 1 diabetes is associated with the superior segmental optic nerve hypoplasia in offspring [6]. In 2010, Tariq *et al.* systematically investigated 2367 children (age 11.1 to 14.4 years) who completed detailed ocular examinations. Results showed that children from diabetic pregnancies had significantly thinner inner and outer macular thickness and macular volume compared with nondiabetic pregnancies [7].

*Pax6* is a member of the paired box family of the highly evolutionally conserved transcription factor [8]. It is a key regulator of eye formation in vertebrates. At the early stage of eye development, *Pax6* expression is located in the presumptive lens ectoderm, lens placode, lens vesicle and optic vesicle [9]. During later eye development, *Pax6* is found in lens, epithelia of conjunctiva, cornea, and in the neural retina [10]. Hill *et al.* [11] and Grindley *et al.* [9] found that mutations of *Pax6* gene, in Homozygous Small eye mice, lack eyes and exhibit central nervous system defects. In humans, heterozygous mutations in *Pax6* have been found in patients with aniridia [12], iris hypoplasia, corneal opacification, autosomal dominant keratitis and isolated foveal hypoplasia [13,14]. These studies clearly showed the importance of *Pax6* in eye development. GDM-induced eye malformation might be related to the mutation of *Pax6*. The increased rate of GDM-induced eye malformation in embryos has raised the need for therapy to improve fetal outcomes.

Proanthocyanidins, a group of abundant natural phenolic compounds in grape seeds, have attracted increasing attention in the fields of nutrition and medicine [15]. Numerous studies have showed that proanthocyanidins exhibit a wide range of effects, including anti-inflammatory [16], anti-arthritic [17], anti-cancer [18], *etc*. In addition, some researches had demonstrated that an increase in oxidative stress and a decrease in anti-oxidative defense in women with GDM were related to perinatal outcomes [19]. Reactive oxygen species (ROS) can impair embryonic defense and induce embryo maldevelopment [20]. Previous studies had also demonstrated that proanthocyanidins can alleviate oxidative stress [21,22] and attenuate diabetes [23,24]. However, limited research has reported the protective effect of proanthocyanidins on eye malformation of embryo. Edaravone, a positive control drug, is a potent scavenger of hydroxyl radicals and attenuates diabetes-induced oxidative damage [25]. Therefore, in this study, we decided to study whether proanthocyanidins can ameliorate the eye malformation of embryos induced by high glucose in chick embryos.

## 2. Experimental Section

### 2.1. Animals and Treatments

Fertilized eggs (South China Agricultural University, China) were incubated in an incubator (Grumbach, Germany) at 37 °C with 60% humidity. To induce hyperglycemia, a window was made in the blunt end of fertilized chicken eggs and 0.2 mmol/egg glucose solution (GLU, Sigma–Aldrich, St. Louis, MO, USA) was injected into the air sac of embryos at embryo development day (EDD) 1. The chicken eggs in control group (CON) were treated with chicken saline solution (0.72% NaCl) at EDD 1. Meanwhile, chick eggs of proanthocyanidins (grape seed extract, 95%, Jianfeng Natural Product R&D Co., Ltd., Tianjin, China) groups were treated with two doses of proanthocyanidins: 1 nmol/egg (ProL) and 10 nmol/egg (ProH) at EDD 0 and 0.2 mmol/egg glucose at EDD 1. Chick eggs of positive control groups were treated with 0.1 nmol/egg edaravone (EDA) at EDD 0 and 0.2 mmol/egg glucose at EDD 1. After treatment, all eggs were further incubated for 36 h or 96 h and then harvested for analysis. All experiments using chick embryos were performed according to the guidelines of the Jinan University Institutional Animal Care and Use Committee.

### 2.2. Mortality, Abnormality, Body Weight and Somite Number Measurements

Embryos of each group were sampled at EDD 3.5. Mortality, abnormality, body weight and somite number measurements were made. Percentage mortality was measured by the number of dead *vs.* all embryos. Rate of eye malformation was assessed by embryos with abnormal eye development *vs.* surviving embryos. Embryos were removed from their eggs and weighed. Somite numbers were also assessed.

### 2.3. Eye Glucose Measurements

Embryonic eye were isolated from embryos of each group to measure eye glucose concentration at EDD 3.5. Eye glucose concentration was measured using a glucose oxidase-coupled spectrophotometric assay kit according to the manufacturer’s instructions (Sigma Chemical Co., St. Louis, Mo, USA).

### 2.4. Histological Analysis

Eye damage was assessed by histological examination in the embryos. At EDD 5, all surviving embryos were harvested and immediately fixed in 4% Paraformaldehyde. Whole embryos were photographed using stereomicroscope (Olympus MVX10) to assess eye malformation. After this, the embryos were immersed in 4% paraformaldehyde for 3 d before paraffin embedment. The paraffin sections were sliced at 5 μm and were processed for hematoxylin-and-eosin (HE) staining.

### 2.5. Measurements of MDA Contents, SOD and GSH-PX Activities and ROS Generation Ratio

Lipid peroxidation in embryo eyes of EDD 3.5 was determined by measuring thiobarbituric-acid-reactive substances (TBARS) with a commercial MDA kit (Nanjing Jiancheng Institute of Biotechnology, Nanjing, China). The activities of total SOD and GSH-PX were measured according the guide of commercial kits (Nanjing Jiancheng Institute of Biotechnology, Nanjing, China). ROS generation ratio was detected with 5 μmol 2′,7′-dichlorofluorescein-diacetate (DCFH-DA, Sigma-Aldrich, St. Louis, Mo, USA).

### 2.6. Quantitative PCR

For quantitative PCR analysis, the dissected eyes from EDD 3.5 embryos were placed in a lysate solution and homogenized. Total RNA was isolated using an RNeasy kit (Qiagen, Hilden, Germany) and reverse-transcribed using SuperScript III Reverse Transcriptase (Invitrogen, Carlsbad, CA, USA). Quantitative PCR was Green. Forward and reverse primers (spanning at least 2 exons) were made using Ensembl genome browser and Primer3 software. Gene expression was determined relative to a calibrator and normalized to the housekeeping gene β*-actin* using the standard curve method and taking primer efficiencies into account. Forward and reverse primers were as follows: *Pax6*: F: 5′-GCTATGACACCTACAC-3′, R: 5′-ACTTGAACTGGAACTG-3′; *Glut1*: F: 5′-TCTCTGTCGCCATCTTCTCG-3′, R: 5′-TGGTGAGGCCAGAATACAGG-3′; β*-actin*: F: 5′-TACCTTCAACTCCATCA-3′, R: 5′-CTCCAATCCAGACAGA-3′.

### 2.7. Western Blotting Analysis

Embryo eyes of EDD 3.5 were separated and lysed in lysis buffer (Beyotime Institute of Biotechnology, Haimen, China) on ice for 10 min. After centrifugation at 12,000 *g* for 15 min, protein content of supernatant was determined with Pierce BCA protein assay kit (Thermo Fisher Scientific, Westminster, MD, USA) to ensure equal sample loading. Protein lysates were separated in 12% SDS-PAGE and blotted onto nitrocellulose membrane (Amersham Biosciences, Piscataway, NJ, USA). Proteins were detected using monoclonal antibody anti-PAX6 diluted 1:1000 (DSHB, USA) and visualized using anti-mouse IgG conjugated with horseradish peroxidase (HRP) and Pierce ECL Western Blotting Substrate (Thermo Fisher Scientific, Westminster, MD, USA) as the substrate of HRP.

### 2.8. Statistical Analysis

Experimental values were given as means ± SD. The statistical analysis between two groups would be evaluated with Student’s unpaired *t*-test. Statistical analysis of the data among multi-groups was performed using the SPSS 18.0 statistical software. Two-way analysis of variance (ANOVA) was applied to analyze for differences in data of biochemical parameters among the different groups, followed by Dunnett’s significant post-hoc test for pairwise multiple comparisons. Differences were considered as statistically significant at * *p* < 0.05, ** *p* < 0.01.

## 3. Results

### 3.1. Proanthocyanidins Ameliorate High Glucose-Induced Eye Malformation in Chick Embryos

**Figure 1:**
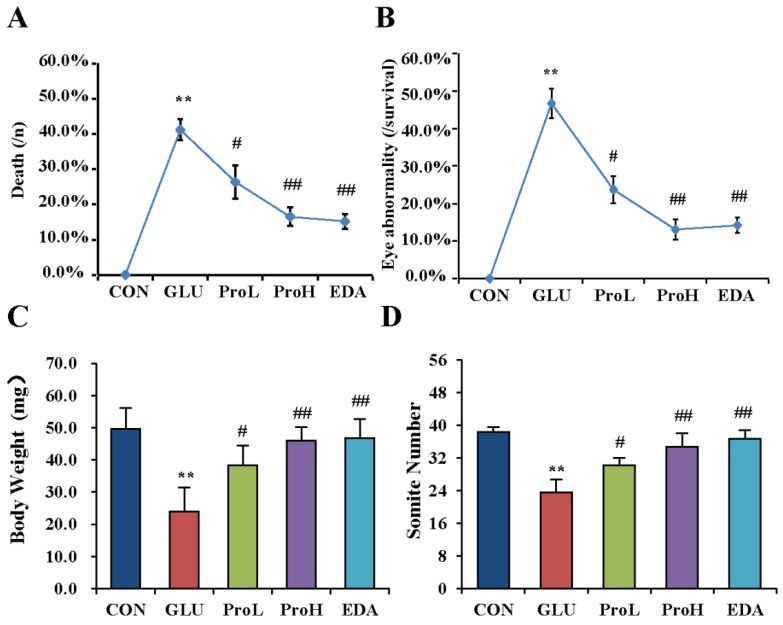
Proanthocyanidins ameliorated high-glucose-induced eye malformation in chick embryo. Death rate (**A**), eye malformation rate (**B**), body weight (**C**) and somite number (**D**) of EDD 3.5 embryos were measured. Morphology images of EDD five embryos. Values were expressed as mean ± SD in each group (*n* = 10). ** *p* < 0.01 *vs.* control, ^#^ *p* < 0.05, ^##^ *p* < 0.01 *vs.* glucose. CON: control, GLU: high glucose, ProL: 1 nmol/egg proanthocyanidins, ProH: 10 nmol/egg proanthocyanidins, EDA: edaravone.

**Figure 2:**
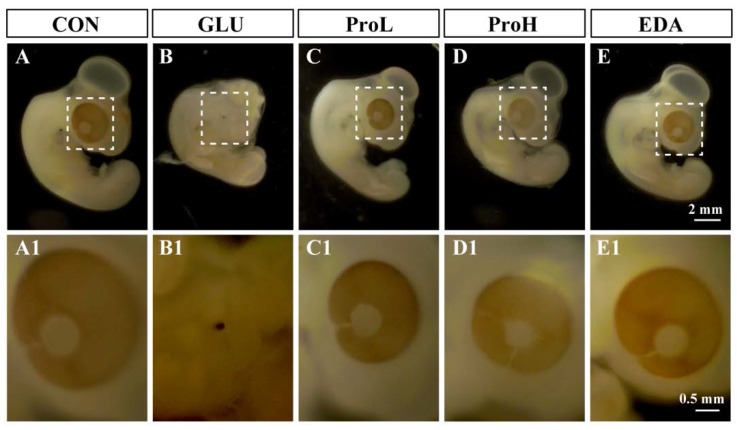
Morphology images of embryo eye development. Stereoscopic microscope measurement of embryo eyes. Scale bars of (**A**–**E)**: 2 mm. Scale bars of (**A1**–**E1)**: 0.5 mm. CON: control, GLU: high glucose, ProL: 1 nmol/egg proanthocyanidins, ProH: 10 nmol/egg proanthocyanidins, EDA: edaravone.

**Figure 3:**
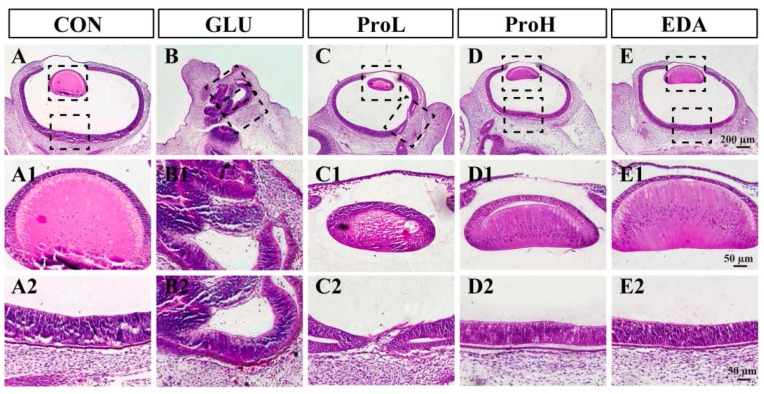
Histological examination of sections from embryo eye. Hematoxylin and eosin (H&E) staining of embryo eyes section. Scale bars of (**A**–**E**): 200 μm. Scale bars of (**A1**–**E2)**: 50 μm. CON: control, GLU: high glucose, ProL: 1 nmol/egg proanthocyanidins, ProH: 10 nmol/egg proanthocyanidins, EDA: edaravone.

To investigate the effect of proanthocyanidins on hyperglycemia-induced eye malformation, different doses of proanthocyanidins (1 and 10 nmol/egg) were injected into to the high-glucose-exposed chick embryos at EDD 0. We found that proanthocyanidins could significantly lower the death rate and decrease the hyperglycemia-induced malformation rate of eyes from 46.7% to 25.7% (ProL) and 13.1% (ProH), respectively (Figure 1A). Edaravone, a well-known antioxidant, could also prevent high glucose-induced malformation. Both doses of proanthocyanidins could significantly recover body weight and somite numbers of chick embryos at EDD 3.5. Meanwhile, edaravone showed similar effect when compared with proanthocyanidins (Figure 1B,C). In morphology analysis, we observed that the embryonic eye was well developed in the control group (Figure 2); the cells were dispersed regularly, and the structures such as lens and retina were clearly distinguished (Figure 3). However, hyperglycemia produced severe abnormal development of chick eye presented as microphthalmos (Figure 2B). The lens size was decreased and the optic cup was smaller. The cells were loosened and disordered; the lens and retina were obscurely formed (Figure 3B). As shown in Figure 2 and Figure 3, ProL and ProH could restore this abnormality. Edaravone also improved the incidence of microphthalmos caused by hyperglycemia.

### 3.2. Proanthocyanidins have No Effects on Glucose Concentration in High Glucose-Treated Eye of Chick Embryos

**Figure 4:**
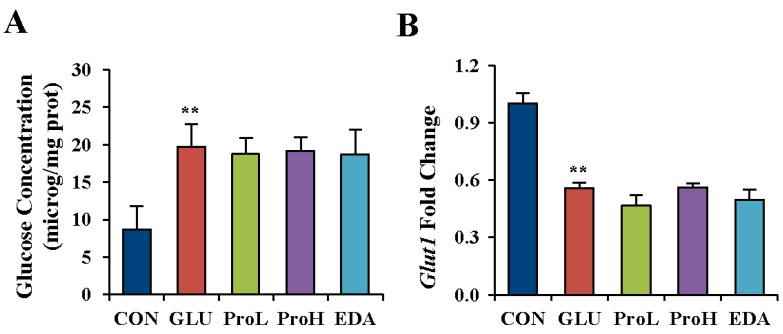
Proanthocyanidins had no effect on glucose concentration in high-glucose-treated eye of chick embryo. Glucose concentration (**A**) and GLUT1 gene expression (**B**) were detected in the eye of EDD 3.5 chick embryo. Values were expressed as mean ± SD in each group (*n* = 10). ** *p* < 0.01 *vs.* control. CON: control, GLU: high glucose, ProL: 1 nmol/egg proanthocyanidins, ProH: 10 nmol/egg proanthocyanidins, EDA: edaravone.

Glucose concentration of eye was determined in EDD 3.5 embryo to further determine the effect of high glucose. Results showed that the injection of 0.2 mmol/egg glucose raised glucose concentration in the eyes of EDD 3.5 embryos, which confirmed that the leading effect was hyperglycemia on embryonic eye malformation. However, the increased glucose content could not be reduced by both proanthocyanidins and edaravone (Figure 4A). Glucose transporters facilitate the transport of glucose across the plasma membrane of a cell, and Glucose transporter 1 (*Glut1*) is one of the major *Gluts* existing in the eye. *Glut1* was measured after high glucose treatment and agents’ administration. Consistently, the gene expression of *Glut1* was decreased in the eye of hyperglycemia-treated embryos. Neither proanthocyanidins nor edaravone recovered their expression (Figure 4B).

### 3.3. Proanthocyanidins Restore the Expression of Eye Development Marker in the Eye of Chick Embryos

**Figure 5:**
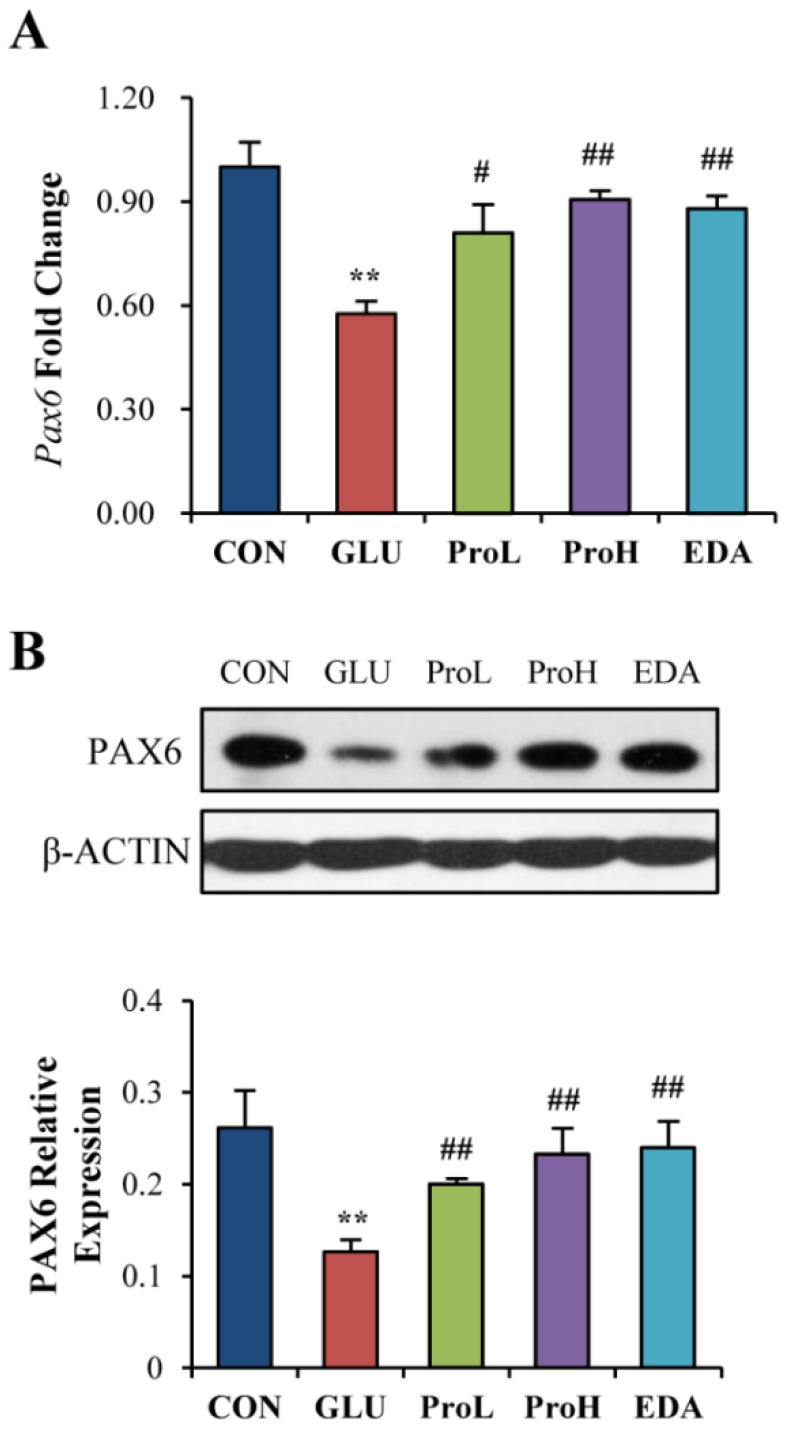
Proanthocyanidins restored the expression of eye development marker in the eye of chick embryo. *Pax6* gene and protein levels (**A** and **B**) were determined in the eye of EDD 3.5 chick embryos. Values were expressed as mean ± SD in each group (*n* = 10). ** *p* < 0.01 *vs.* control, ^#^ *p* < 0.05, ^##^ *p* < 0.01 *vs.* glucose. CON: control, GLU: high glucose, ProL: 1 nmol/egg proanthocyanidins, ProH: 10 nmol/egg proanthocyanidins, EDA: edaravone.

*Pax6* is a master transcriptional gene that regulates the formation of the optic vesicle, optic cup, lens placode and retina and mutation of *Pax6* causes eye malformation [26,27]. To better understand the mechanism of how proanthocyanidins protected the malformation induced by high glucose, we further examined *Pax6* expression in the eye of EDD 3.5 chick embryo. As shown in Figure 5, high glucose inhibited both gene and protein expression of *Pax6*. The two doses of proanthocyanidins could elevate the expression of *Pax6*. Consistently, edaravone could also increase *Pax6* expression. We had also detected some other molecular markers of eye development. High glucose also inhibited the expression of *Six3* [28] and *Otx2* [29]. However, proanthocyanidins could not restore them. In addition, high glucose did not affect the expression of *Mitf* [30], *Rx1* [31] and *Chx10* [32] (Supplemental Figure). These results indicated that proanthocyanidins treatment prevented eye malformation mainly by restoring *Pax6* expression.

### 3.4. Proanthocyanidins Ameliorated the Oxidative Stress State Induced by High Glucose in the Eye of Chick Embryos

**Figure 6:**
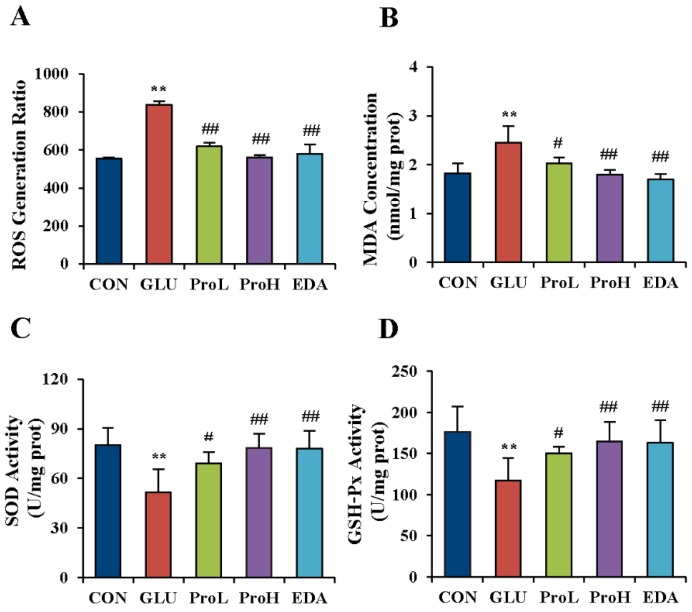
Proanthocyanidins ameliorated the oxidative stress state induced by high glucose in the eye of chick embryo. ROS generation ratio (**A**), MDA level (**B**), SOD (**C**) and GSH-PX (**D**) activities were measured in the eye of EDD 3.5 chick embryos. Values were expressed as mean ± SD in each group (*n* = 10). ** *p* < 0.01 *vs.* control, ^#^ *p* < 0.05, ^##^ *p* < 0.01 *vs.* glucose. CON: control, GLU: high glucose, ProL: 1 nmol/egg proanthocyanidins, ProH: 10 nmol/egg proanthocyanidins, EDA: edaravone.

It has been reported that hyperglycemia could increase ROS production and blocking ROS production could ameliorate the effects of hyperglycaemia [33]. In order to further evaluate the recovering effect of proanthocyanidins on high glucose-induced eye malformation, we investigated the anti-oxidation ability of proanthocyanidins in the eyes of chick embryos at EDD 3.5. As shown in Figure 6A. ROS generation was significantly increased after glucose treatment. The injection of proanthocyanidins and edaravone respectively in the high-glucose-treated embryo before glucose exposure had slowed down the generation of ROS. MDA, an indicator for lipid peroxidation induced by ROS, was also elevated with glucose administration. Both proanthocyanidins and edaravone could prevent MDA accumulation in the eyes (Figure 6B). The activities of free oxygen radical scavenger enzymes were investigated by determining SOD and GSH-Px activities. Results showed that SOD and GSH-Px were decreased in the eyes of high glucose-treated embryos (Figure 6C,D). Both proanthocyanidins and edaravone recovered the activities of these enzymes.

## 4. Discussion

Offspring involved in gestational diabetes mellitus have a higher risk of being born with congenital malformations, including cardiovascular defects, CNS abnormality and skeletal dysplasia [34]. In this study, we used chick embryo to establish a GDM model to study the protective effect of proanthocyanidins on eye development of embryos. When chick embryos were exposed to 0.2 mmol/egg glucose on EDD 1, the glucose concentration of embryo eye was elevated on EDD 3.5. *Glut1* is identified as the major glucose transporters in CNS and facilitate the transport of glucose across the plasma membrane of a cell. The *Glut1*, which is expressed in the eye, mediates the cellular uptake of glucose [35]. In the current study, high glucose condition resulted in an increase of intracellular glucose concentration and a decrease in *Glut1* gene expression in the embryo eyes on EDD 3.5. The level of *Glut1* was not significantly different at the early stage [36]. This increased intracellular glucose concentration at the early stage which was sustained until EDD 3.5. It is important to note that similar downregulation of glucose transporters in response to high glucose condition is seen *in vivo* and *in vitro* [37,38,39]. Conversely, glucose deprivation could cause a rapid and sustained increase in *Glut1* mRNA and protein [40,41]. The mechanisms for the regulation of *Glut1* by glucose availability are still not clear. We speculated that the downregulation of *Glut1* might be a feedback regulation of the high level of intracellular glucose, which can reduce the glucose uptake. After high glucose exposure, the weight of embryos decreased, which was identified as developmental retardation. Microphthalmos and anophthalmia was also found in chicken embryos after high glucose exposure. These results indicated that the GDM model was successfully established. In order to prevent this embryonic disease, two doses of proanthocyanidins—1 nmol/egg (ProL) and 10 nmol/egg (ProH)—were injected into the air sac before glucose treatment. We found that proanthocyanidins could ameliorate high glucose-induced death rate, eye malformation rate and body weight loss in chick embryos. However, proanthocyanidins could not reverse the elevated glucose concentration in the embryo eye. These data suggested that the protective effect of proanthocyanidins was not related to hypoglycemic effect.

*Pax6* is a highly evolutionally conserved transcription factor related to eye formation. Furthermore, the mutation of *Pax6* causes aniridia and induces diabetes [14]. Therefore, we proposed that *Pax6* is one of the targets in high glucose-induced eye malformation. PCR and Western blot results showed that *Pax6* expression was disturbed in the high glucose-treated embryo eyes. However, proanthocyanidins and edaravone could recover the high glucose-induced *Pax6* down-regulation. Therefore, the high glucose-induced eye malformation might be related to *Pax6* down-regulation.

It has been reported that hyperglycemia could promote ROS production, while preventing ROS production could compensate the effects of hyperglycemia [33]. During the embryogenesis, insulin is not yet produced by the conceptus and could not be obtained from maternal circulation [42]. Pancreatic insulin does not appear in chick embryos until EDD 3.5 to EDD 4 [43]. Disturbances in metabolism during early pregnancy are responsible for defective organogenesis in diabetic pregnancy [44]. Embryos have a high level of oxygen consumption and are exceptionally vulnerable to oxidative damage. However, their antioxidant defense mechanisms are not well-developed. Oxidative stress has long been linked to diabetes or GDM [45,46,47]. In this study, high glucose treatment led to excess ROS generation in the eyes of chick embryos. ROS is thought to exert its deleterious effects primarily by damaging virtually all classes of biomolecules including DNA, protein and lipid, leading to cell death [48]. It has been proposed that oxidative damage contributes to the development of diabetic retinopathy [49]. It had been demonstrated that *Pax6* was susceptible to oxidative stress. PAX6 protein could be easily oxidized and excluded from the nucleus of stressed corneal epithelial cells, with concomitant loss of corneal epithelial markers [50]. These phenomena proved that high glucose-induced suppression of *Pax6* might be related to oxidative stress. Proanthocyanidins have been reported to exhibit antioxidant effect [15,21,51]. Consistently, proanthocyanidins alleviated the oxidative stress state, restored *Pax6* expression and prevented the high-glucose-induced eye malformation in the chick embryo, having similar effects of edaravone.

## 5. Conclusions

In this study, we successfully established a GDM model to study the protective effect of proanthocyanidins on GDM-induced embryo eye malformation. We showed that high glucose-mediated suppression of *Pax6* is a critical target of proanthocyanidins. However, the effect of GDM on the developing embryo is complex. A lot of work needs to be done to obtain a complete understanding of the phenomena.

## Supplementary Files

Supplementary File 1
