# Supplementary material for: Proanthocyanidins Prevent High Glucose-Induced Eye Malformation by Restoring Pax6 Expression in Chick Embryo
Source: Nutrients. 2015 Aug 7;7(8):6567–81. doi: 10.3390/nu7085299 (PMC4555138; doi:10.3390/nu7085299)
Supplement: Supplementary File 1 [file nutrients-07-05299-s001.docx]

Supplemental Figure

**
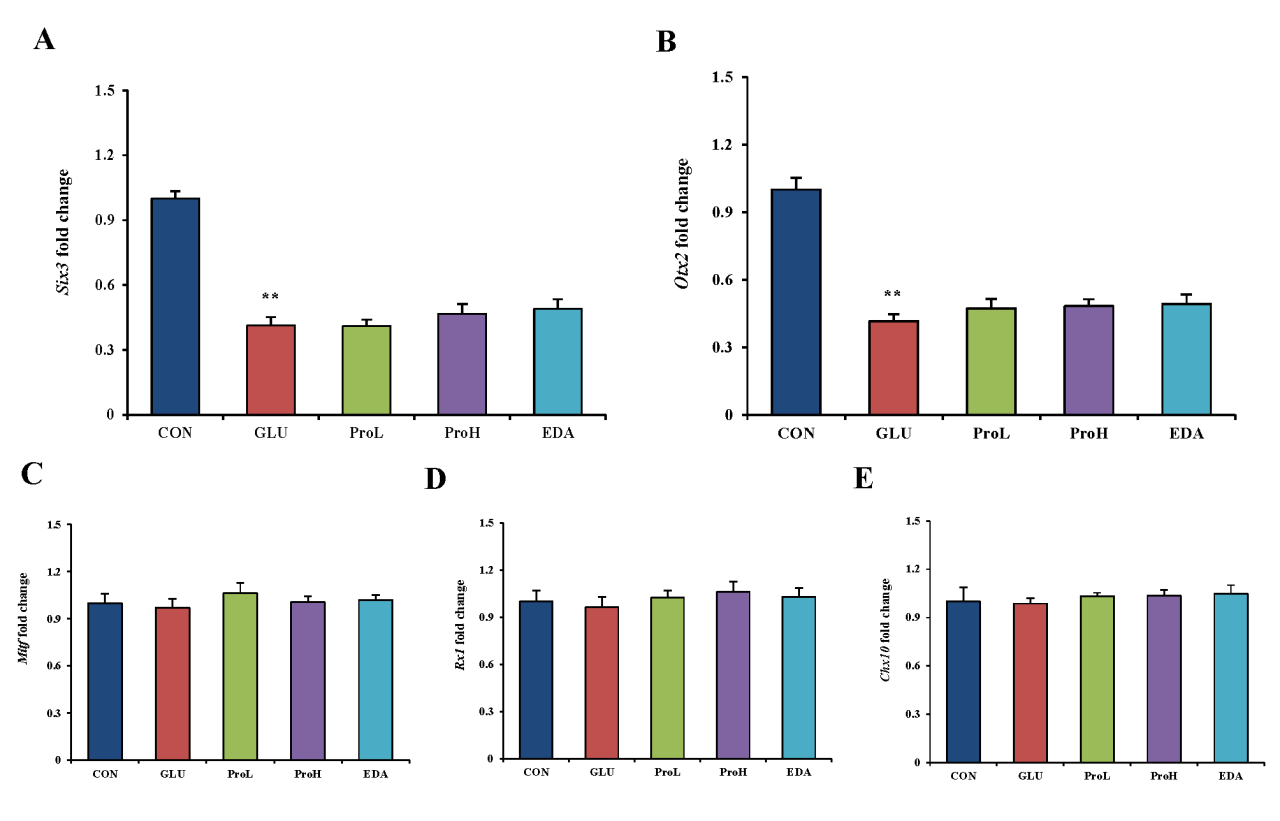
**

**Figure S1.** The effect of proanthocyanidins on the expression of eye development marker in the eye of chick embryo. *Six3* (**A**), *Otx2* (**B**), *Mitf.* (**C**), *Rx1* (**D**) and *Chx10* (**E**) gene levels were determined in the eye of EDD 3.5 chick embryos. Values were expressed as mean ± SD in each group (*n* = 10). ** *p* < 0.01 *vs.* control. CON: control, GLU: high glucose, ProL: 1 nmol/egg proanthocyanidins, ProH: 10 nmol/egg proanthocyanidins, EDA: edaravone.

© 2015 by the authors; licensee MDPI, Basel, Switzerland. This article is an open access article distributed under the terms and conditions of the Creative Commons Attribution license (http://creativecommons.org/licenses/by/4.0/).
